# MEF-TBN: Multi-exposure image fusion approach based on a three-branch network for feature extraction and color enhancement

**DOI:** 10.1371/journal.pone.0357156

**Published:** 2026-09-21

**Authors:** Chunmeng Wang, Wenxiang Zhang, Quan Zhang, Siyi Zhou

**Affiliations:** School of Computer Engineering, Jinling Institute of Technology, Nanjing, China; Islamia University of Bahawalpur: The Islamia University of Bahawalpur Pakistan, PAKISTAN

## Abstract

High dynamic range (HDR) imaging is crucial in fields like photography and medical image processing. Traditional multi-exposure fusion (MEF) methods often struggle to simultaneously preserve local and global features as well as chrominance information. To address this issue, we propose MEF-TBN, a three-branch network consisting of a context aggregation attention network (CAAN) branch for local feature extraction, a transformer branch for global dependency modeling, and a color enhancement branch for natural color restoration. Specifically, the CAAN and transformer branches generate low-resolution weight maps based on local and global features of luminance components, and a merging module is proposed to refine and enhance the local and global features with several merger blocks, and then estimate high-resolution weights by adopting the guided filtering for upsampling operation. Meanwhile, the color enhancement branch is capable of learning the inherent correlation between luminance and chrominance components. Experimental results show that compared with 9 representative MEF methods, our method achieves optimal performance on most image feature-based metrics including AG, EI and SF, as well as all human perception-oriented metrics such as Q^CB^, VIF and NIQE, and ranks second in the image structural similarity-based metric MEF-SSIM. In particular, compared with the state-of-the-art transformer-based method SwinFusion, our method achieves average performance improvements of 11.5%, 19.8%, 2.2% and 1.6% on Q^CB^, VIF, NIQE and MEF-SSIM metrics, respectively. Both quantitative and qualitative experiments conducted on three MEF datasets with different input exposure numbers demonstrate that the proposed method achieves superior performance than the other methods.

## 1. Introduction

High dynamic range (HDR) imaging technology [1] has been widely used in many fields, such as photography, medical image processing, remote sensing monitoring and so on. However, one single low dynamic range (LDR) image cannot contain all the information in overexposed and underexposed regions so as to lose useful details and colors [2]. Fortunately, the multi-exposure fusion (MEF) technology provides an effective approach by fusing different exposed images to generate the high dynamic range (HDR) image, which preserves details and color information in the overexposed and underexposed regions.

The current deep learning based MEF methods usually have the problems of local or global detail lost and color distortion because they do not fully consider the effects of human visual system (HVS) for subjective quality improvement. Firstly, the convolutional neural network (CNN) based models have obvious advantages on extracting local features, but have disadvantage for modeling global long-distance dependency due to the small receptive fields. Secondly, the transformer based methods have advantage on long-distance dependency modeling by window-based self-attention mechanisms, but have shortcomings in dealing with local features and capturing fine textures, resulting in slightly blurred outputs and insufficient details compared with CNN-based methods. Besides, the transformer based methods tend to concentrate on salient regions while neglecting informative background areas with low saliency, leading to an imbalance between global representation and local detail preservation.

How to combine CNN and transformer is very important to improve dynamic range and preserve details of the fused image. Although existing hybrid CNN-Transformer methods attempted to integrate local and global feature extraction, they still have several disadvantages. Firstly, most of these methods adopted common convolutional modules without multi-scale context aggregation attention enhancement mechanism, which restricts the ability to capture useful local details. Secondly, they commonly relied on simple concatenation or complex attention-based mechanism to fuse CNN and transformer outputs, lacking a suitable and effective fusion mechanism to fully integrate complementary information. Thirdly, most methods only designed the fusion strategy for the luminance component but adopting simple weighted summation for each chrominance component, which caused color distortion in the over-exposed or under-exposed regions of the fused image. Therefore, it is necessary to restore the color information in each chrominance component with specialized color enhancement mechanism to improve the human visual perception quality.

To overcome the above shortcomings, we propose a three-branch network for the MEF task to preserve both local and global features and enhance color information, called MEF-TBN, including context aggregation attention network (CAAN) branch, transformer branch and color enhancement branch. With such a collaborative three-branch structure, our method effectively alleviates detail loss and color distortion that remain challenging for existing hybrid CNN-Transformer fusion methods. The main contributions are as follows:

(1) The context aggregation attention mechanism is proposed to extract multi-scale local features with one CAAN branch instead of the simple CNN, and the transformer branch is proposed to capture long-distance dependency features with multi-head self-attention (MSA) mechanism for restoring global dynamic range.
(2) A merger module is proposed to integrate and enhance features with several merger blocks more effectively, and guided filtering for joint upsampling (GFU) is proposed to generate the original resolution weight maps from the low-resolution version for maintaining edge consistency and improving efficiency.
(3) The color enhancement branch is proposed to optimize the appearance by learning the relationship between color and luminance in natural images, and the visual perception quality of fused image is significantly improved with vivid and natural colors.

## 2. Related works

A variety of MEF algorithms have been proposed [3], which are mainly divided into traditional methods and deep learning based methods. The traditional methods are mainly based on spatial domain or transform domain.

The spatial domain based methods usually apply fusion strategy with each pixel [4] or image patch [5]. Liu et al. [6] proposed a pixel-wise method by using dense scale invariant feature transform descriptor as a measure, so as to calculate the weights for fusion. However, the pixel-wise methods do not consider the global dynamic range retention and cause brightness inversion. Ma et al. [7] proposed a patch-wise method based on structural patch decomposition, and fused multi-exposure images by decomposition strategy optimization, but the patch-based methods usually introduce halo artifacts on the edge regions between patches.

The transform domain based methods usually transform the input images from spatial domain to another domain, then apply a fusion strategy on the domain coefficients, and finally convert the fused coefficients back to the spatial domain. For example, Burt et al. [8] first proposed a pyramid decomposition strategy to complete the MEF task. Mertens et al. [9] proposed a Laplacian pyramid decomposition method based on [8] with the weight maps measured by contrast, saturation, and well-exposedness. Li et al. [10] used guided filtering to decompose input images into a base layer and a detail layer, and then merged each layer by weighted average. Wang et al. [11] proposed a multi-scale exposure fusion method in YUV space with a simple detail enhancement component, which is difficult to recover enough details when dealing with complex HDR scenes. The transform domain based methods often adopt complex transformation and fusion strategies with higher complexity and lower robustness in complex scenes.

The deep learning methods have achieved remarkable results for the MEF task. Prabhakar et al. [12] proposed the first method to implement MEF task with convolutional neural network (CNN), and optimized the network with the unsupervised loss function MEF-SSIM [13]. However, the method has shortcomings of detail lost and color distortion because of its simple network model. Ma et al. [14] proposed MEF-Net by using context aggregation network to extract multi-scale features. Similar with CNN, it is unable to capture the long-distance dependency information, thus the overall fusion effect is not satisfactory. MEF-CAAN [15] addressed the issue of detail restoration in extremely exposed areas with the channel attention module and multi-scale context aggregation, but it still cannot deal with long-distance dependency features. Qu et al. [16] designed an encoder that combines a CNN module with a transformer module to extract long-distance dependency features, which proved the effectiveness to extract local features with CNN and global features with transformer. However, there are three disadvantages. Firstly, the simple CNN module cannot extract multi-scale features without contextual aggregation attention mechanism. Secondly, the simple enhance block was used for combining features from the CNN module and the transformer module without more effective feature merger mechanism. Thirdly, color distortions still occur in underexposed and overexposed areas because chrominance components are ignored. Besides, several advanced transformer-based fusion networks have been developed for general image fusion, which are also suitable for MEF task. Ma et al. [17] proposed a fusion network SwinFusion that utilizes intra-domain self-attention and inter-domain cross-attention to model long-range global dependencies based on Swin Transformer. Although it achieved powerful global representation ability, the heavy transformer structure and complex cross-attention leaded to over-smoothing artifacts, insufficient local detail preservation and high computational cost. Zhao et al. [18] proposed the dual-branch CNN-Transformer architecture CDDFuse to capture low-frequency global contexts and high-frequency local details, but it has severe color distortion issues in extremely under-exposed or over-exposed regions. Nevertheless, it is originally designed for multi-modality fusion but cannot well adapt to the MEF task. DPE-MEF [19] used a network including one feature extraction module and one color enhancement module, but it ignores the importance of different channels, so that it cannot extract enough feature information. Li et al. [20] proposed to enhance colors of overexposed and underexposed areas by learning to estimate and correct color distortion with deformable convolutions, but it is difficult to accurately estimate the distortion direction when the local texture is lack of gradient information, resulting in local color inconsistency. MEF-LUT [21] encoded the weights of exposures with a one-dimensional lookup table to improve efficiency, but the fusion quality is very unsatisfactory. All the above methods are unsupervised and do not require ground-truth fused images. Xu et al. [22] and Yang et al. [23] both proposed generative adversarial network (GAN) based methods that incorporated adversarial game into the network training to implicitly fulfill feature extraction, feature fusion and image reconstruction. However, the fusion quality by the GAN-based methods depends on the dataset that are manually designed or derived from other methods.

Recently, diffusion models have been used in the field of high dynamic range (HDR) imaging, such as Li et al. [24] and Chen et al. [25], which usually transform the MEF task into an image inpainting problem and adopt diffusion priors to restore an extreme exposed image to a well-exposed image. However, the lack of high-quality diffusion prior may affect the quality of image restoration, and the iterative sampling of the diffusion process is quite time-consuming.

Some unified fusion models were proposed to apply for various image fusion tasks including MEF. For example, U2Fusion [26] utilized feature gradients to maintain the similarity between the fused image and the source images, but the fusion results are prone to cause color distortion and detail blur in certain local areas due to gradient sensitivity. GIFNet [27] proposed a three-branch architecture for multiple fusion tasks, but it has problems of local detail lost and color distortion because feature extraction of the main task is easy to be affected by other auxiliary tasks. However, the multi-branch modeling architecture has been demonstrated effective for image enhancement and fusion. For example, Çiftçi [28] proposed an adaptive multi-branch feature fusion framework for low-light image enhancement, jointly modeling luminance and chrominance information, edge and texture structures, frequency and domain information, and so on. Our method also adopts a three-branch architecture for multi-exposure image fusion.

Besides, some multi-exposure image fusion [29] and multimodal image fusion [30,31] models were proposed for medical image registration or image fusion to enhance image quality, but these methods are mainly aimed at medical image processing.

## 3. Methods

**Fig 1:**
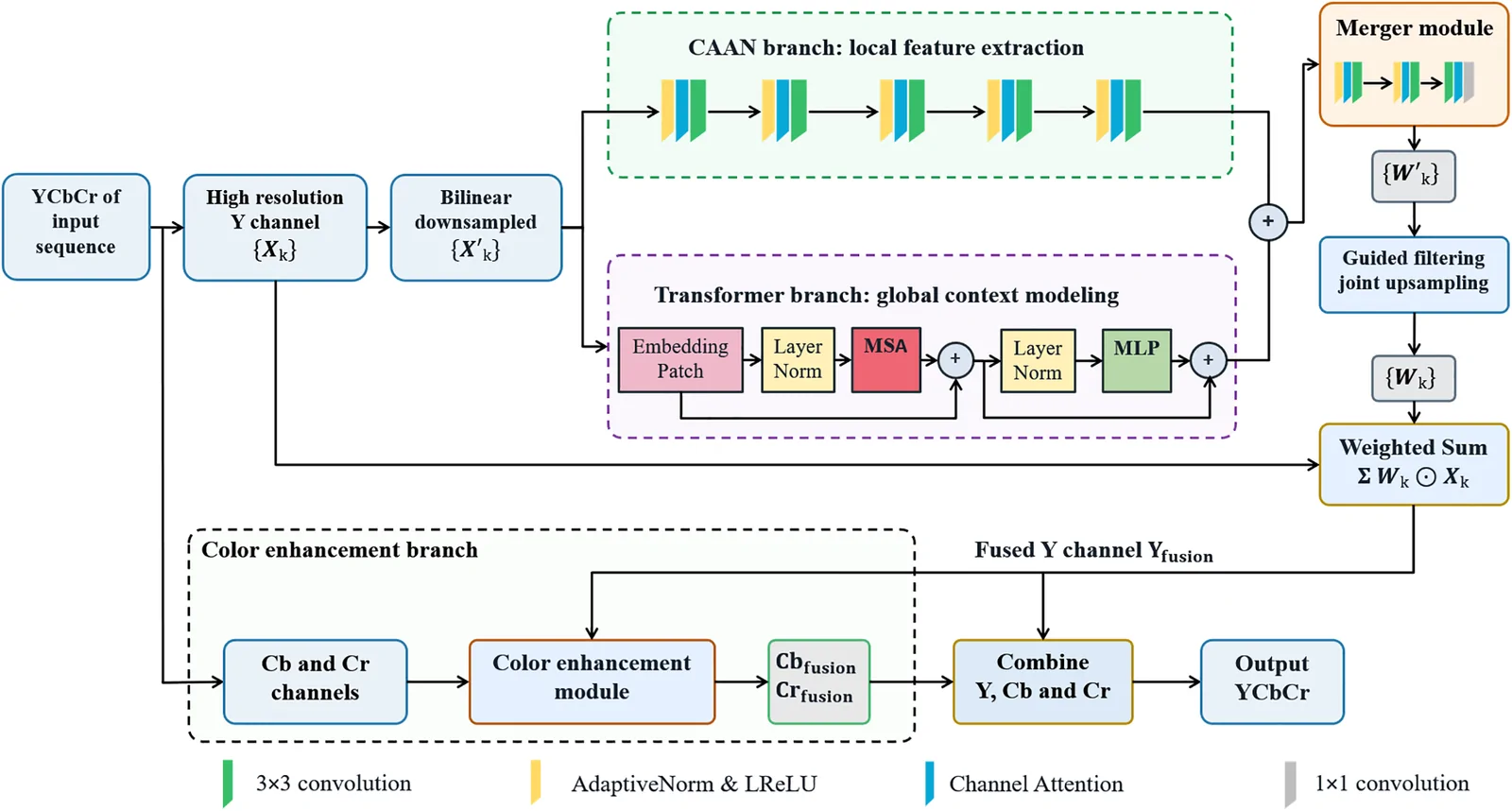
Overall structure diagram of MEF-TBN.

The input multi-exposure images of our MEF-TBN need to be well aligned. MEF-TBN is processed in YCbCr color space that is converted from RGB color space for luminance details preserving and color enhancement separately. The overall structure of the MEF-TBN is shown in Fig 1.

The input images are denoted by $\left\{X_{k}\right\},\;k=\text{1},\;\ldots ,\;K$, and K is the number of input images. $\left\{X_{k}\right\}$ is resized to the low-resolution version $\left\{X_{k}^{\prime }\right\}$ with bilinear downsampling to reduce the complexity of the model as follows:


$$\begin{array}{c} X_{k}^{\prime }=BDS\;\left(X_{k}\right),\;{X_{k}\in R^{\text{3}\times H\times W}\;\text{and}\;X}_{k}^{\prime }\in R^{\text{3}\times H^{\prime }\times W^{\prime }} \end{array}$$ (1)

where BDS represents the operation of bilinear downsampling with the default scale 2. H and W are height and width of the original input image, respectively. $H^{\prime }$ and $W^{\prime }$ are height and width of the downsampled image, respectively. The bilinear downsampling is adopted to reduce redundant computational burden and GPU memory consumption, which is critical for efficient network training and testing. Though bilinear downsampling operation can cause to lose high-frequency details, the subsequent guided filtering joint upsampling (GFU) module can compensate and reconstruct the high-frequency information with the high-resolution original images $\left\{X_{k}\right\}$ as guided to reduce the detail information loss, which has been proven effective in the MEF-Net [14].

Because human visual system (HVS) is more sensitive to the changes of structural and luminance information mainly contained in the luminance channel Y, the low-resolution version of Y channel $\left\{X_{k}^{\prime }\right\}$ is imported into both CAAN and transformer branches to extract local and global features. The output weight maps of CAAN branch and transformer branch are $\left\{W_{k}^{\prime }(C)\right\}$ and $\left\{W_{k}^{\prime }(T)\right\}$, respectively. Then the merger module integrates $\left\{W_{k}^{\prime }(C)\right\}$ and $\left\{W_{k}^{\prime }(T)\right\}$ and further generates low-resolution weight maps $\left\{W_{k}^{\prime }\right\}$. Both $\left\{X_{k}\right\}$ and $\left\{W_{k}^{\prime }\right\}$ are input as guided to generate high-resolution weight maps $\left\{W_{k}\right\}$ for fusion with guided filtering for joint upsampling, and the fused Y channel Y_fusion_ is obtained by weighted summation of $X_{k}$ and $W_{k}$. For the chrominance channels (Cb and Cr) with dominant color information, the color enhancement branch is adopted for fusing each chrominance channel to preserve and enhance color information. The color enhancement module of the branch also uses Y_fusion_ and input YCbCr images as reference to generate each fused chrominance component (Cb_fusion_ and Cr_fusion_).

### 3.1. Contextual aggregation attention network branch

The CAAN branch mainly aims to extract local feature information from the source images. CAAN was first proposed in [15] based on convolutional neural network (CNN) and channel attention mechanism. The network uses dilated convolution to increase the receptive field without changing the spatial resolution. It aggregates context information at multi-scales by stacking convolutional layers with different dilation rates for better feature extraction. By learning the weights between different channels, the channel attention mechanism enhances the attention to more important channel features and suppresses less important channel features.

The network of CAAN branch contains six layers, including five convolutional layers and one output layer as shown in Fig 1. All the layers maintain feature maps as the same resolution with the input image. The five convolutional layers all adopt the 3×3 kernel with dilation rates as 1, 2, 4, 8 and 1, corresponding to the receptive field 3×3, 7×7, 15×15, 31×31 and 33×33, respectively. The output layer utilizes the 1×1 kernel with dilation rate as 1. The output channel width is set to 24 for all the convolutional layers to balance quality and efficiency. An adaptive normalization operation and the activation function LReLU with a negative slope of 0.2 are applied following each convolutional layer. The adaptive normalization can adjust parameters according to different input data flexibly, and it can reduce the gradient explosion problem for training faster formulated as follows:.


$$\begin{array}{c} F_{an}\left(T_{F}\right)=\alpha_{n}T_{F}+\beta_{n}IN\left(T_{F}\right) \end{array}$$ (2)

where $\alpha_{n},\;\beta_{n}\in R$ are learnable weight parameters, $T_{F}$ is the intermediate feature representation, and IN(·) represents the instance normalization operation.

The channel attention mechanism is introduced to enhance the feature extraction ability of the network. In the channel attention mechanism module, the squeeze operation is performed on the feature maps, and the global average pooling operation is used to encode the channel features into global features, formulated as follows:


$$\begin{array}{c} z_{c}=F_{sq}(T_{F})=\frac{\text{1}}{H^{\prime }\times W^{\prime }}\sum_{i=\text{1}}^{H^{\prime }}\sum_{j=\text{1}}^{W^{\prime }}T_{F}(i,j) \end{array}$$ (3)

where i and j represent the pixel coordinates, $F_{sq}$ is the squeeze operation, and $T_{F}$ is the intermediate feature input with the dimension number C.

The excitation operation is applied to the global features, which consists of two convolutional layers and two activation functions with LReLU. The excitation operation enables the network to learn the relationship between channels and generate the weights of different channels. The excitation operation is calculated as follows:


$$\begin{array}{c} s_{c}=F_{ex}\left(z_{c},\omega \right)=\sigma \left(g\left(z_{c},\omega \right)\right)=\sigma \left(\omega_{\text{2}}\sigma \left(\omega_{\text{1}}z_{c}\right)\right) \end{array}$$ (4)

where $\omega_{\text{1}},\;\omega_{\text{2}}\in R^{\frac{C}{r}\times C}$ denotes that the dimension is $\frac{C}{r}\times C$, r is the scaling factor, σ denotes the LReLU activation function, and then the operation $F_{scale}()$ is performed and each feature map is multiplied by its assigned weight to obtain the feature map processed by the channel attention mechanism formulated as follows:


$$\begin{array}{c} {F_{c}=F}_{scale}(T_{F},\;s_{c})=T_{F}\cdot s_{c} \end{array}$$ (5)

Finally, the output layer generates the weight maps of the CAAN branch $\left\{W_{k}^{\prime }(C)\right\}$ using 1×1 convolution formulated as follows:


$$\begin{array}{c} {W_{k}^{\prime }(C)=F}_{\text{c}onv\;\text{1}\times \text{1}}(F_{c}) \end{array}$$ (6)

### 3.2. Transformer branch

**Fig 2:**
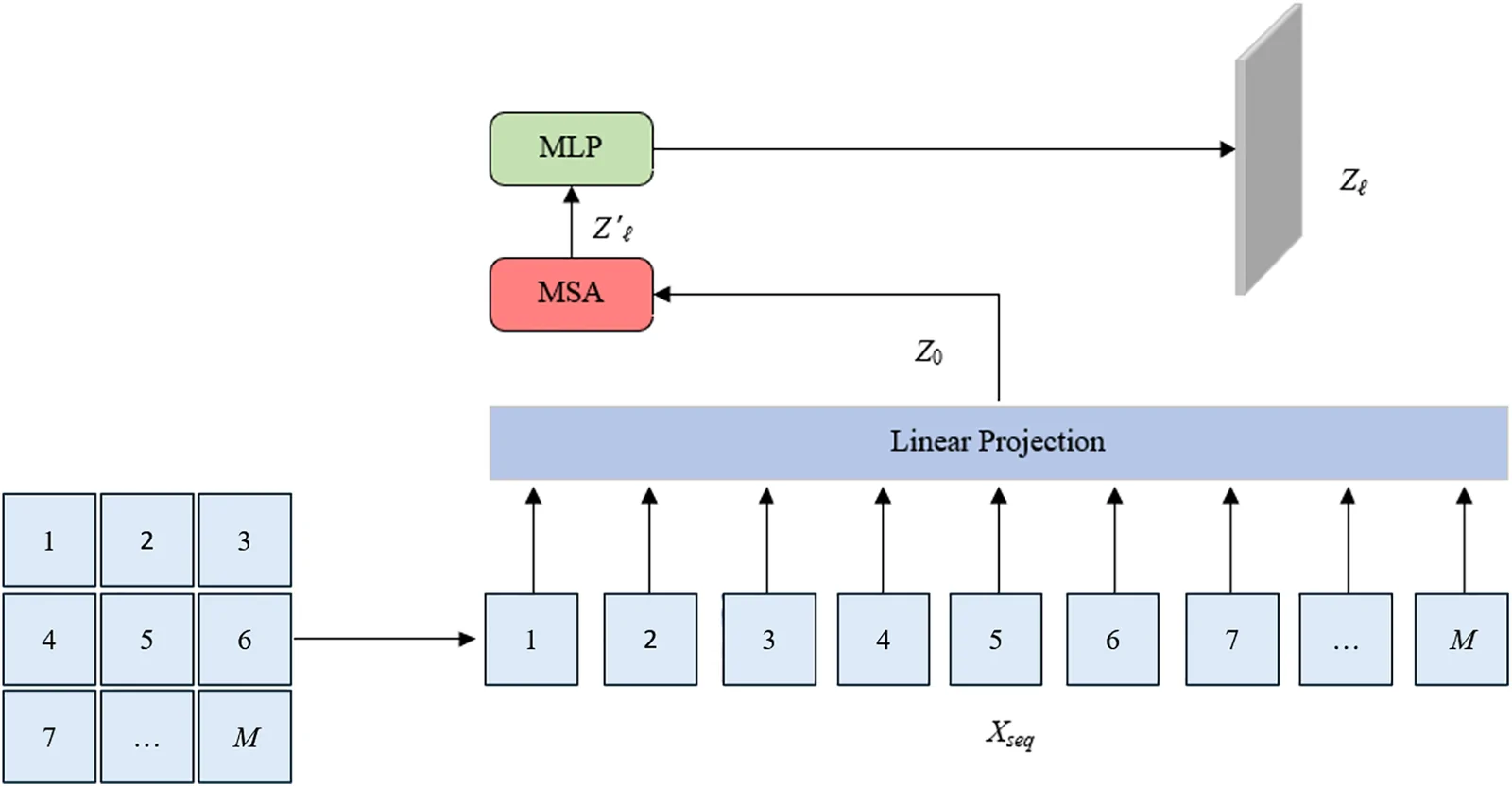
The process of the transformer branch.

The CAAN branch can extract local feature information of the input images, but it has low ability to model global long-distance dependency as the increasement of network depth. The transformer branch is able to overcome the shortcoming and has advantage on long-distance dependency modeling. Inspired by Qu et al. [16] based on Visual Transformer (ViT), we first resize each input image to the size of 256×256 to meet the transformer input requirement, and then divide it into M patches with the size of P×P to build the flattened sequence $X_{seq}=\left\{X_{m}\right\},(m=\text{1},\;\text{2},\ldots ,M)$. The parameter settings are consistent with those in the Qu et al. [16], with the default values of P and M are 16 and 256, respectively. The modules of transformer branch is shown in Fig 2.

The transformer branch encodes each patch in position to obtain the encoded sequence $Z_{\text{0}}$ by the linear projection as the following formula:


$$\begin{array}{c} Z_{\text{0}}=\left[X_{class};X_{\text{1}}E;X_{\text{2}}E;\cdots ;X_{M}E\right]+E_{pos} \end{array}$$ (7)

where $X_{class}$ represents the marking operation of image blocks, $X_{M}$ represents each marked image block, $E\in R^{P^{\text{2}}\times D},E_{pos}\in R^{(M+\text{1})\times D}$, D represents the dimension of the embedding vector with the default value 256.

The layer normalization is used to normalize the sequence features $Z_{\text{0}}$ to stabilize the training process and accelerate convergence, and then multi-head self-attention (MSA) is used to model global dependency between image patches. It allows each patch to directly interact with other long-distance patches by the self-attention mechanism. The process is formulated as follows:


$$\begin{array}{c} Z_{\text{𝓁}}^{\prime }=MSA\left(LN\left(Z_{\text{𝓁}-\text{1}}\right)\right)+Z_{\text{𝓁}-\text{1}},\text{ 𝓁}=\text{1}\ldots L \end{array}$$ (8)

where $Z_{\text{𝓁}-\text{1}}$ and $Z_{\text{𝓁}}^{\prime }$ represent the input and output of the 𝓁-th MSA block, respectively. MSA represents the operation of multi-head self-attention (MSA) and LN represents the operation of layer normalization.

After the same layer normalization for normalizing the output of the 𝓁-th MSA block $Z_{\text{𝓁}}^{\prime }$, the multilayer perceptron (MLP) is used to further nonlinearize the transformer features to prevent training overfitting, which contains two fully connection layers and a dropout layer. This process is formulated as follows:


$$\begin{array}{c} Z_{\text{𝓁}}=MLP\left(LN\left(Z_{\text{𝓁}}^{\prime }\right)\right)+Z_{\text{𝓁}}^{\prime },\text{ 𝓁}=\text{1}\ldots L \end{array}$$ (9)

where $Z_{\text{𝓁}}^{\prime }$ and $Z_{\text{𝓁}}$ respectively represent the input and output of the 𝓁-th MLP block. L represents the number of MSA or MLP blocks, and the default L is 12, with the same setting as Qu et al. [16]. The residual connection is used after each MSA and MLP, so that even if the update effect of MSA and MLP is not good, the original image features are still preserved through the residual information to avoid performance degradation. Finally, the output weight maps of feature block $Z_{L}$ of the transformer branch $\left\{W_{k}^{\prime }(T)\right\}$ is formulated as follows:


$$\begin{array}{c} W_{k}^{\prime }(T)=MLP\left(LN\left(Z_{L}^{\prime }\right)\right)+Z_{L}^{\prime } \end{array}$$ (10)

### 3.3. The merger module

In our fusion mechanism, the local and global features are first combined by element-wise addition, and then refined by several stacked convolution-based merger blocks. Different from the cross-attention fusion SwinFusion [17], our strategy retains the original representation of local and global features as much as possible, and then uses convolution to refine and enhance the fused features. This mechanism avoids heavy computation, over-smoothing and information distortion caused by complex attention mechanisms, making it more stable and effective for features fusion.

The merger module is proposed to integrate the low-resolution weight maps for local feature extracted by the CAAN branch $W_{k}^{\prime }(C)$ and the low-resolution weight maps for global feature generated by the transformer branch $W_{k}^{\prime }(T)$ formulated as follows:


$$\begin{array}{c} W_{k}^{\prime }=MG\left(W_{k}^{\prime }(C),W_{k}^{\prime }(T)\right) \end{array}$$ (11)

where $W_{k}^{\prime }$ represents the k-th low-resolution weight map of the output, MG represents the network of merging module for the features from the two branches.

Our network of merger module contains three merger blocks, and each merger block contains two 3×3 convolution layers and one adaptive normalization layer with the LReLU activation function, as shown in Fig 1. The first convolution layer in each merger block is responsible for integrate the features extracted by the two branches, and the second convolution layer can abstract the integrated features into higher level semantics features. The normalization and LReLU activation function can make the network propagate more stably and the training converge faster. The output layer is a 1×1 convolution, resulting in the low-resolution weight maps.

Our feature extraction network branches, including both CAAN and transformer branches, are processed in low-resolution space to improve efficiency. The guided filtering for joint upsampling is applied to upscale $\left\{W_{k}^{\prime }\right\}$ to the original resolution weight maps $\left\{W_{k}\right\}$, which takes the source image sequence $\left\{X_{k}\right\}$, low-resolution version $\left\{X_{k}^{\prime }\right\}$ and low-resolution weight maps $\left\{W_{k}^{\prime }\right\}$ as input as follows:


$$\begin{array}{c} W_{k}=GFU(X_{k},X_{k}^{\prime },W_{k}^{\prime }) \end{array}$$ (12)

where GFU is the operation of guided filtering for joint upsampling [15].

For guided filtering, there is a linear relationship between the input guided map $I_{i}$ and the output map $q_{i}$, which is expressed as follows:


$$\begin{array}{c} q_{i}=a_{k}I_{i}+b_{k},\;\forall i\in \omega_{k} \end{array}$$ (13)

where $a_{k}$ and $b_{k}$ denote constant parameters with a local square window with radius r=1. Guided filtering introduces the concept of noise, that is, the output image $q_{i}$ is the denoised image of the input image $p_{i}$. The gap between the output of the fitted function $q_{i}$ and the true value $p_{i}$ is minimized as follows:


$$\begin{array}{c} E(a_{k},b_{k})=\sum_{i\in \omega_{k}}\left({\left(a_{k}I_{i}+b_{k}-p_{i}\right)}^{\text{2}}+\epsilon a_{k}^{\text{2}}\right) \end{array}$$ (14)

where ε is the regularization parameter with $\varepsilon ={\text{10}}^{-\text{4}}$.

Finally, we generate the k-th normalized weight map ${\hat{W}}_{k}$ as follows:


$$\begin{array}{c} {\hat{W}}_{k}=\frac{W_{k}}{\sum_{k=\text{1}}^{K}W_{k}} \end{array}$$ (15)

where $W_{k}$ is the k-th weight map. Then the Y channel of the fused image Y_fusion_ is obtained by weighted summation of ${\hat{W}}_{k}$ and the Y channel of the k-th input image $X_{k}^{Y}$ as follows:


$$\begin{array}{c} Y_{f}=\sum_{k=\text{1}}^{K}X_{k}^{Y}\;⨂\;{\hat{W}}_{k} \end{array}$$ (16)

where ⨂ denotes the Hadamard product, and $Y_{f}$ is the Y channel of fused image.

### 3.4. Color enhancement branch

Rich and vivid color information is also important to improve the visual perception quality of the fused image. However, most existing MEF methods first convert input images from RGB into YCbCr color space, and then focus on the luminance component (Y), but perform simple weighted fusion for each chrominance component (Cb or Cr), which can lead to serious color distortion and lost if not all the input images are well-exposed. To solve the problem, we design a color enhancement branch inspired by [19], which aims to learn the mapping relationship between brightness and color in the same scene.

The input of our color enhancement module in this branch is the fused Y channel Y_fusion_ by the previous two branches and two input images. In order to fully consider the color information under extreme exposure condition, the most over-exposed and under-exposed images in each sequence are selected as the two input images as follows:


$$\begin{array}{c} \left[{Cb}_{f},{Cr}_{f}\right]=F_{e}(X_{max},X_{min},Y_{f},\varepsilon ) \end{array}$$ (17)

where $F_{e}$ is the color enhancement module, $X_{max}$ and $X_{min}$ represent the most over-exposed and under-exposed images in the sequence, respectively, $Y_{f}$ is the Y channel of the fused image Y_fusion_, and ε represents the parameters to be learned in the network. ${Cb}_{f}$ and ${Cr}_{f}$ represent the two output fused chrominance component Cb_fusion_ and Cr_fusion_, respectively.

**Fig 3:**
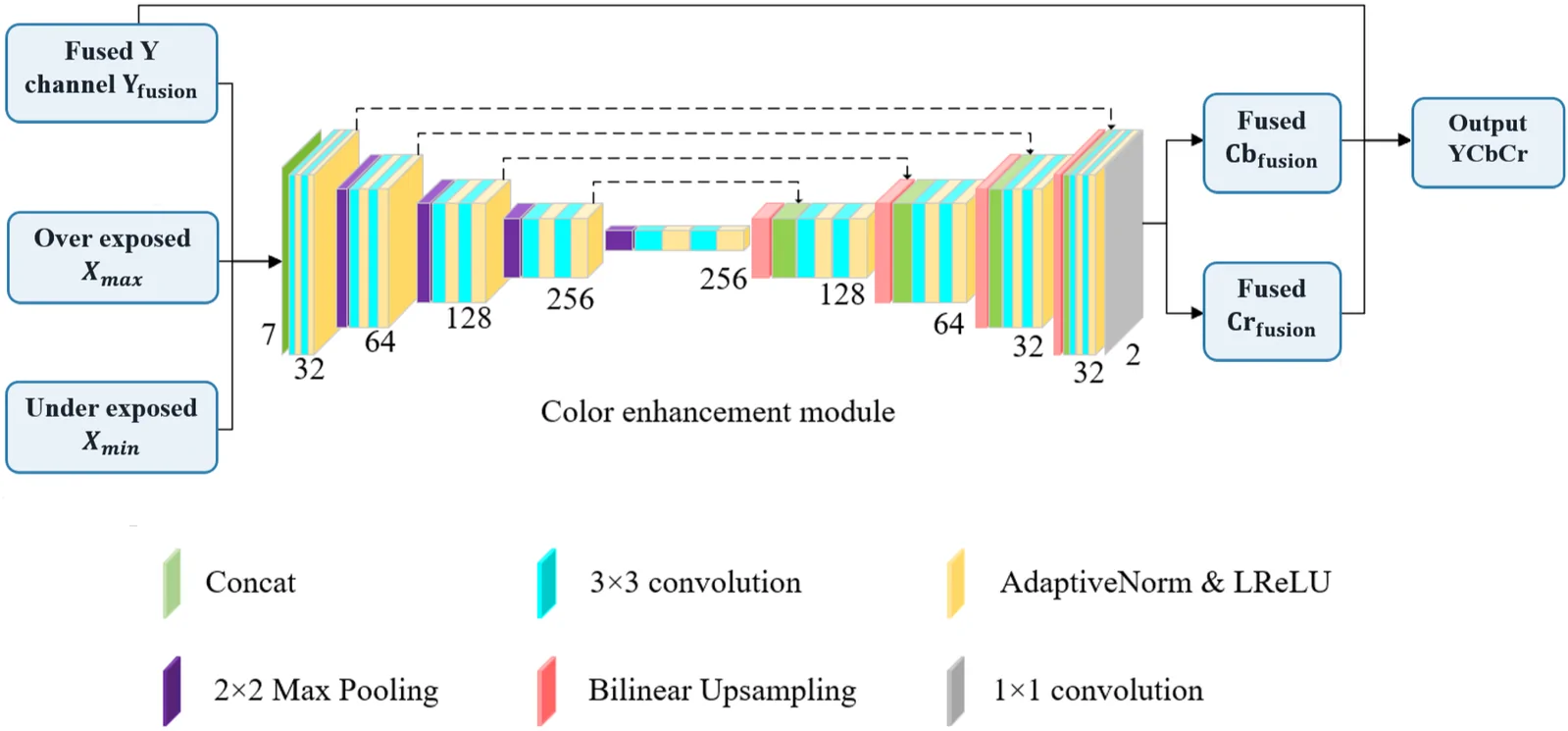
The network of color enhancement branch.

The network of color enhancement module contains a symmetric encoder and decoder structure based on a fully convolutional network as shown in Fig 3. Firstly, the encoder receives a 7-channel tensor from the three YCbCr channels of $X_{max}$, the three YCbCr channels of $X_{min}$, and the fused luminance channel $Y_{f}$. The encoder is performed with multiple convolutional blocks and each block contains two 3×3 convolution layers, two adaptive normalization layers with activation function LReLU and one 2×2 max pooling layer. The number of output channels for each layer is set to 32, 64, 128, 256 and 256, respectively. All of them are multiples of 32 to balance the performance and efficiency, and increases to 256 as the size of feature maps shrinking gradually. Then the decoder expands the size of feature maps by bilinear upsampling, and uses the similar network with the encoder, including a concat layer, two convolutions layers, two adaptive normalization layers with LReLU, and connects the corresponding layer with the encoder by skipping connection to refine the features, and a 1×1 convolution layer is applied to generate the output image of the fused chrominance component Cb_fusion_ and Cr_fusion_ as ${Cb}_{f}$ and ${Cr}_{f}$.

Finally, the fused RGB image is converted back from the YCbCr color space based on the fused Y_fusion_ image $Y_{f}$ and the two fused chrominance component images ${Cb}_{f}$ and ${Cr}_{f}$.

The color enhancement branch is necessary because the main fusion branches focus on structural and illumination information, which may weaken or distort color information. This branch effectively preserves color saturation and reduces color distortion, ensuring the fused image has vivid and natural colors consistent with human visual perception.

### 3.5. Loss functions

We adopt two different loss functions for training luminance component and chrominance components, respectively.

For the luminance image, the structural similarity index based MEF metric MEF-SSIM [13] is adopted to train the CAAN branch, the transformer branch and the merging module, which evaluates the quality of the fused image with intensity, contrast and structure information. MEF-SSIM is a no-reference quality metric computed from the input exposure images $\left\{X_{k}\right\}$ without the ground-truth fused image. The loss function has been adopted by some previous unsupervised MEF methods such as DeepFuse [12] and MEF-Net [14]. The metric is more consistent with human visual and can effectively preserve intensity, contrast and structural detail information, which is suitable for our objective of local and global features fusion.

For computing MEF-SSIM metric, each image patch $x_{k}$ is determined by:


$$\begin{array}{c} x_{k}=\left\|x_{k}-\mu_{x_{k}}\right\|\cdot \frac{x_{k}-\mu_{x_{k}}}{\left\|x_{k}-\mu_{x_{k}}\right\|}+\mu_{x_{k}}=\left\|{\tilde{x}}_{k}\right\|\cdot \frac{{\tilde{x}}_{k}}{\left\|{\tilde{x}}_{k}\right\|}+\mu_{x_{k}}=c_{k}\cdot s_{k}+l_{k} \end{array}$$ (18)

where ‖·‖ denotes the ${\text{𝓁}}_{\text{2}}$-norm. $c_{k}=\left\|{\tilde{x}}_{k}\right\|$, $s_{k}=\frac{{\tilde{x}}_{k}}{\left\|{\tilde{x}}_{k}\right\|}$, and $l_{k}=\mu_{x_{k}}$ represent the contrast, structure, and intensity of $x_{k}$, respectively.

The quality measure of each local image patch is determined by:


$$\begin{array}{c} S(\{x_{k}\},y)=\frac{\left(\text{2}\mu_{\hat{x}}\mu_{y}+C_{\text{1}})(\text{2}\sigma_{\hat{x}y}+C_{\text{2}}\right)}{\left(\mu_{\hat{x}}^{\text{2}}+\mu_{y}^{\text{2}}+C_{\text{1}})(\sigma_{\hat{x}}^{\text{2}}+\sigma_{y}^{\text{2}}+C_{\text{2}}\right)} \end{array}$$ (19)

where $\mu_{\hat{x}}$ and $\mu_{y}$ denote the mean intensities of the desired patch and the fused patch, respectively. $C_{\text{1}}$ and $C_{\text{2}}$ are two constants to avoid zero denominators and their default values are both ${\text{1}\text{0}}^{-\text{4}}$. $\sigma_{\hat{x}}$ and $\sigma_{y}$ denote the variances of the patches $\hat{x}$ and y, respectively. $\sigma_{\hat{x}y}$ is the covariance of the patches $\hat{x}$ and y.

The loss of the fused luminance image $Y_{f}$ is obtained by averaging all patches by:


$$\begin{array}{c} Loss\text{1}=-\text{MEFSSIM}(\{X_{k}\},Y_{f})=-\frac{\text{1}}{M}\sum_{i=\text{1}}^{M}S(\{R_{i}X_{k}\},R_{i}Y_{f}) \end{array}$$ (20)

where $R_{i}$ is the i-th patch of the image. M is the number of patches. We aim to minimize the loss function to maximize the MEF-SSIM score and improve the quality of the fused luminance image.

For the two chrominance images, the most over-exposed image $X_{max}$, the most under-exposed image $X_{min}$ and the middle-exposed image $X_{mid}$ are selected from each multi-exposure sequence to train the color enhancement branch. The network models the mapping relationship between color and luminance in the scene according to the brightness and the corresponding chroma of the most over-exposed and under-exposed images, and then infers the desired color information under the luminance of the medium exposure level that can be regarded as reference to supervise the network training, since it possesses natural and reliable chrominance distribution without severe over-exposure or under-exposure distortion. The $L_{\text{1}}$-norm is employed to constrain the deviation between the predicted chrominance and the reference designed as follows:


$$\begin{array}{c} Loss\text{2}={\left\|{Cb}_{f}-{Cb}_{m}\right\|}_{\text{1}}+{\left\|{Cr}_{f}-{Cr}_{m}\right\|}_{\text{1}} \end{array}$$ (21)

where ${\left\|\cdot \right\|}_{\text{1}}$ denotes the $L_{\text{1}}$-norm operation. ${Cb}_{m}$ and ${Cr}_{m}$ represent two chrominance components of the medium exposure level image. The same weights are assigned to the two chrominance components because the significance of the Cb and Cr is equal.

Above all, our separated loss function design for luminance and chrominance channels achieves a reasonable balance between structural detail preservation and color fidelity, which is well validated for our MEF task.

### 3.6. Training

We use the SICE set from Cai [31] as our training dataset including 589 static sequences, and each sequence contains at least 3 exposures. The training dataset can be downloaded on the website https://github.com/csjcai/SICE. These sequences include many kinds of scenes, such as indoor and outdoor, daytime and night, nature and architecture, and so on. The two-stage training strategy is adopted due to the different training data and loss functions for luminance feature extraction and color enhancement. All the sequence images are used to train the CAAN branch, the transformer branch and the merging module, while the most over-exposed, under-exposed and middle-exposed images are selected from each sequence to train the color enhancement branch. During training, the input high-resolution images of the luminance fusion network are bilinearly resized to the default resolution of 512×512 from the original images, and the low-resolution images are bilinearly downsampled to 256×256. The training process was implemented on one NVIDIA GeForce RTX 2080 SUPER GPU of 16GB memory with Python = 3.9.18 and PyTorch = 2.2.1. We adopt Adam optimizer for the two-stage training. The epoch is set to 600 and the batch size is set to 8. The initial learning rate is set to ${\text{1}\text{0}}^{-\text{4}}$, and the decay interval and decay ratio of the learning rate schedule are set to 1000 and 0.1, respectively. The seed control is randomized with a default value 2025. The peak GPU memory usage for training is 6.8GB, with the usage ratio about 43% of the whole memory of 16GB. The training time is about 18 hours for all the epochs. The parameter $r_{L}$ of LReLU is set to 0.2, and the radius r and regularization parameter ε of guided filtering are set to 1 and ${\text{1}\text{0}}^{-\text{4}}$, respectively.

## 4. Experiment results

We compare the proposed method with 9 state-of-the-art methods by both subjective observation and quantitative evaluation, including DEM [11], MEF-Net [14], TransMEF [16], SwinFusion [17], CDDFuse [18], DPE-MEF [19], MEF-LUT [21], UltraFusion [25] and GIFNet [27]. DEM [11] is the representative traditional method without training, and the others are all deep learning based methods. For fair comparison, MEF-Net [14], TransMEF [16], CDDFuse [18] and GIFNet [27] were retrained on the same training dataset SICE [32], and the methods SwinFusion [17], DPE-MEF [19], MEF-LUT [21] and UltraFusion [25] were evaluated using their official public pretrained models. The input of our method can be any resolution and any number of input images, so we firstly conducted comparative testing for K=2 because all these methods can support two input images. Besides, we also conducted comparative testing with the most representative methods including DEM [11], MEF-Net [14], SwinFusion [17] and MEF-LUT [21] for K=3 and K=5, because some MEF methods only support K=2, such as TransMEF [16], CDDFuse [18], DPE-MEF [19], UltraFusion [25] and GIFNet [27]. The dataset MEFB provided by Zhang [33] is used for testing. The sequences of training and testing dataset are listed in S1 Table of the Supporting Information, including 45 sequences for K=2, 15 sequences for K=3 and 15 sequences for K=5.

### 4.1. Subjective observation comparison

**Fig 4:**
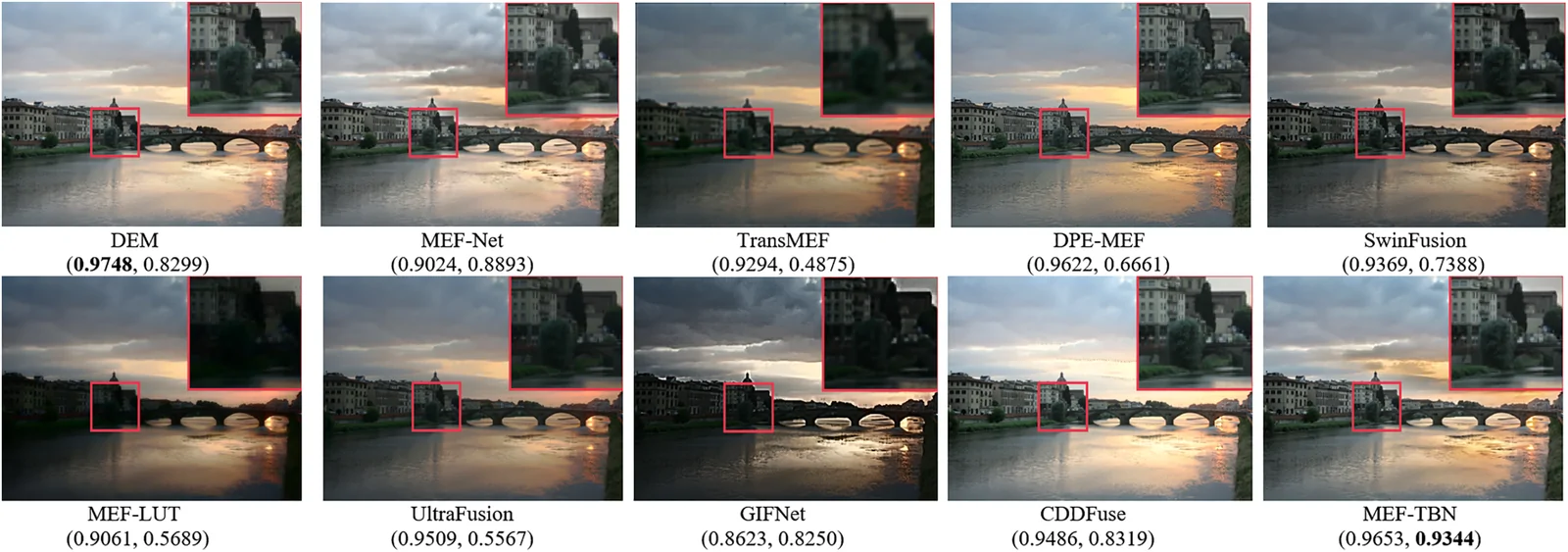
The fusion results of 10 MEF methods on the sequence ‘Arno’, and two metrics (MEF-SSIM, VIF) with maximum scores highlighted in bold.

**Fig 5:**
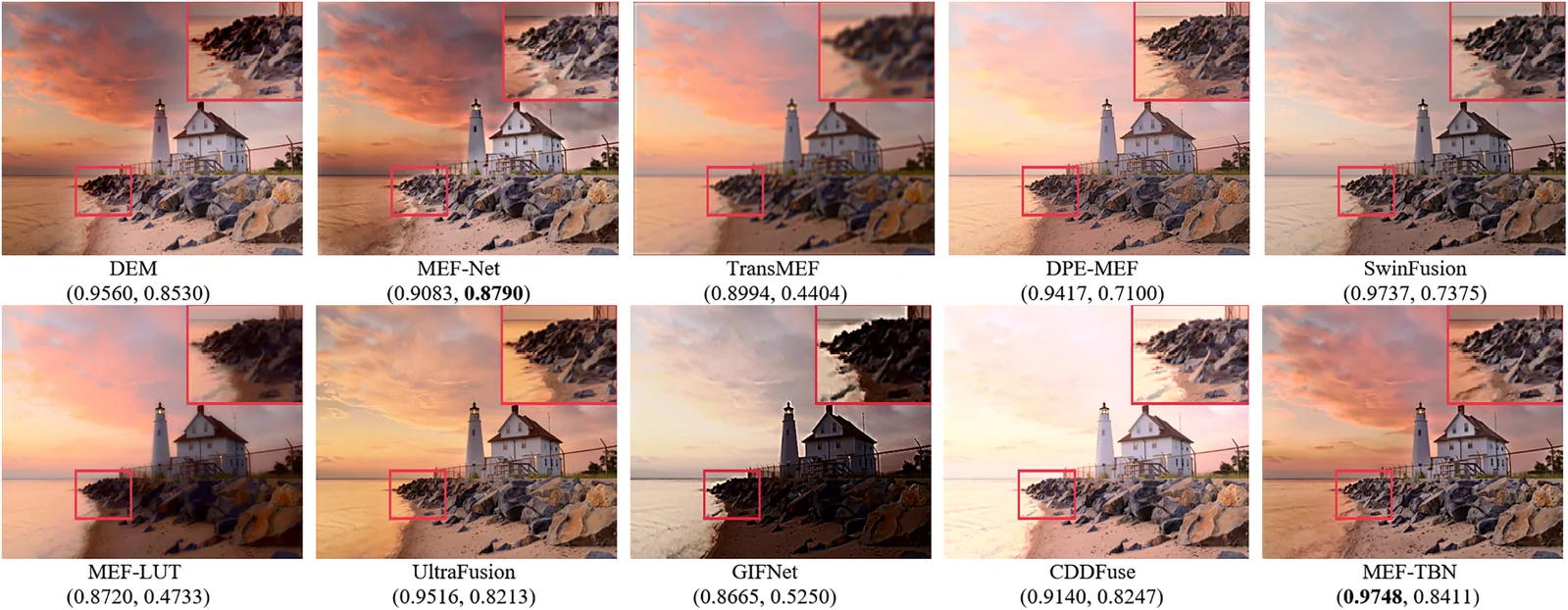
The fusion results of 10 MEF methods on the sequence ‘Lighthouse’, and two metrics (MEF-SSIM, VIF) with maxi-mum scores highlighted in bold.

**Fig 6:**
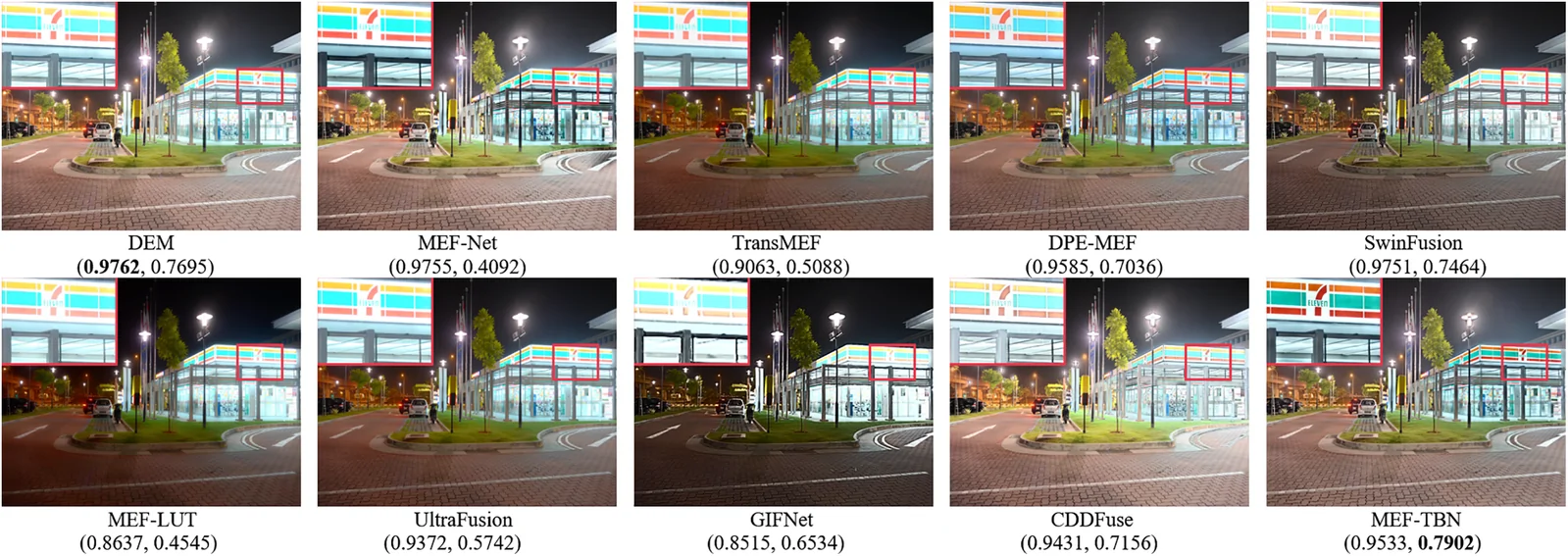
The fusion results of 10 MEF methods on the sequence ‘SevenElevenNight’, and two metrics (MEF-SSIM, VIF) with maximum scores highlighted in bold.

**Fig 7:**
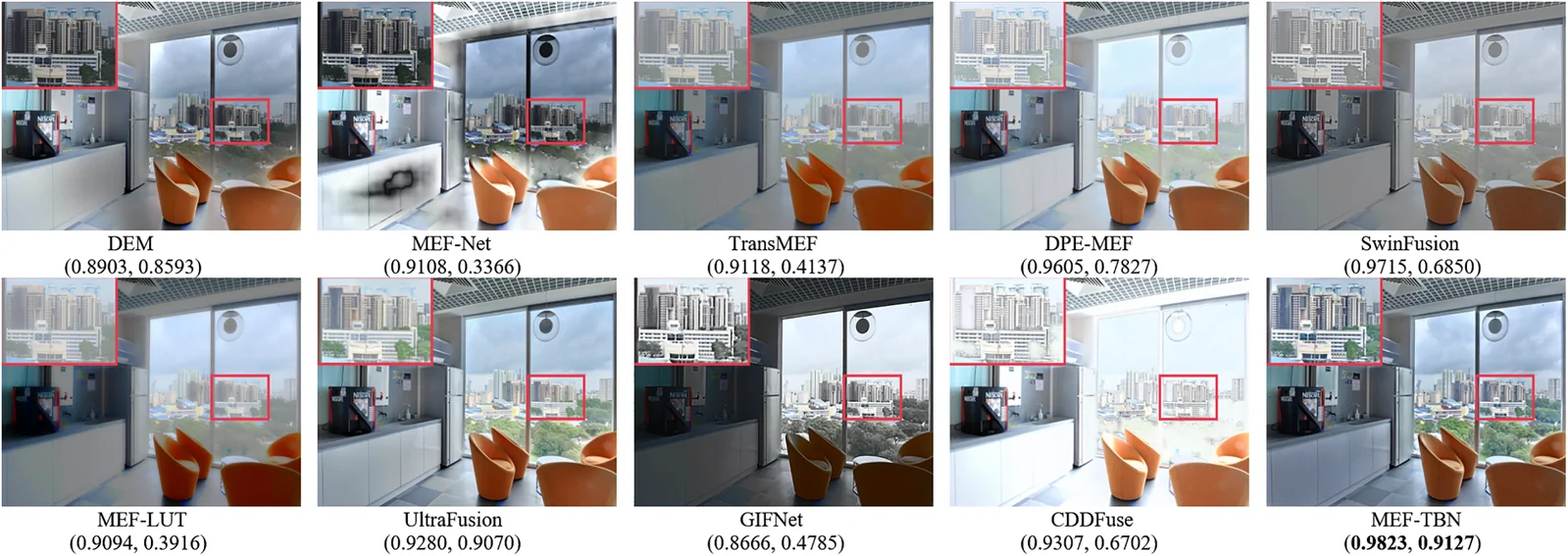
The fusion results of 10 MEF methods on the sequence ‘PantrySmallISO’, and two metrics (MEF-SSIM, VIF) with maximum scores highlighted in bold.

**Fig 8:**
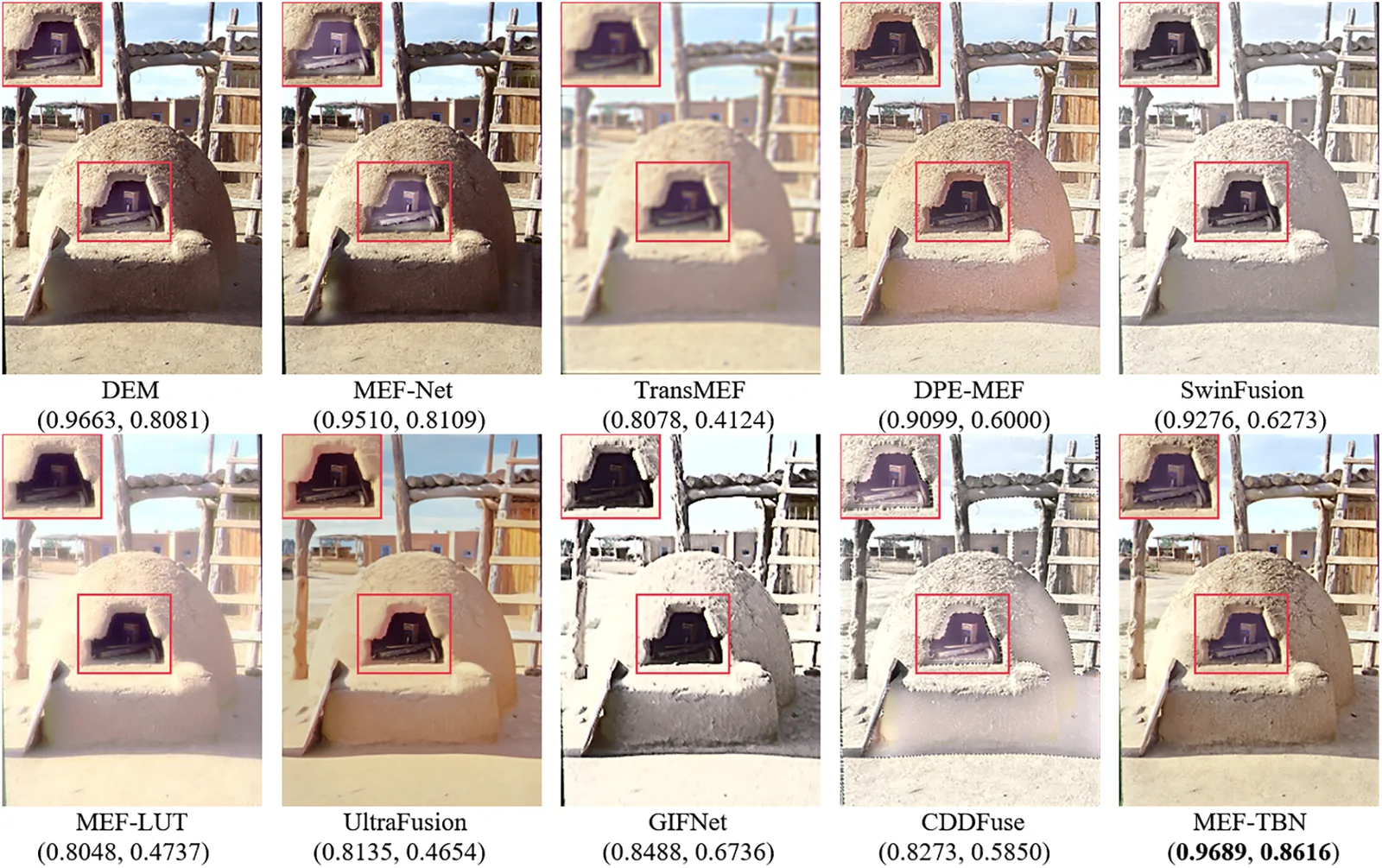
The fusion results of 10 MEF methods on the sequence ‘Igloo’, and two metrics (MEF-SSIM, VIF) with maximum scores highlighted in bold.

The fusion results for the sequences are presented from Figs 4–8, including outdoor natural scenery in Figs 4 and 5, nighttime scene in Fig 6, daytime indoor scene in Fig 7 and outdoor architectural scene in Fig 8.

The results of other methods have insufficient details on the bridge of Fig 4, on the beach of Fig 5, in the store of Fig 6, outside the window of Fig 7 and inside the igloo of Fig 8. The results of DEM [11] and MEF-Net [14] introduce halo artifacts, such as in the Figs 4, 7 and 8. The results of TransMEF [16], MEF-LUT [21] and GIFNet [27] fail to recover the dynamic range and color information with serious loss of details and color distortion, especially in the under-exposed areas, and all the results of TransMEF [16] look significantly blurred with high-frequency details lost. Though DPE-MEF [19] avoids the above issues, it has low contrast and color saturation, such as in the sky of Fig 5 and the scenery outside the window of Fig 7. UltraFusion [25] also has the problem of color distortion, such as in Fig 8. The transformer-based state-of-the-art methods SwinFusion [17] and CDDFuse [18] also have over-smoothing artifacts and color distortion defect, such as in Figs 5, 7 and 8. Our method MEF-TBN still presents clearer details, more natural color reproduction and better exposure consistency. Moreover, we have presented the scores of the representative structural similarity based metric MEF-SSIM [13] and the representative human perception inspired metric VIF [34] for all the results of Figs 4-8 to provide a quantitative comparison. Our method MEF-TBN achieves highest scores of the two metrics for most scenes, which also demonstrates the superiority of our method.

To better support our perceptual evaluations, we have conducted a small-scale user study. Specifically, we invited 8 volunteers (including 4 researchers and 4 non-professional participants) to conduct a blind pairwise preference comparisons. The experiment provided 10 randomly selected testing sequences generated by 10 comparative methods without any labels, and the volunteers were asked to select the image with richer details, better exposure level, more natural colors and better visual quality under the same observation condition. The total selection number is 3600 (45×8×10) and the selection number of each method is up to 720. The statistical results are summarized in Table 1, including total selection number, preference rate (total selection number / 720) and ranking for each method, which shows that our method achieves the highest preference rate 86.1% and ranks first among all methods. UltraFusion obtains the second highest preference rate 73.1%. In contrast, MEF-LUT [21], TransMEF [16] and GIFNet [27] perform poorly in terms of visual perception, with preference rates only 10.8%, 11.9% and 26.9%, respectively.

**Table 1:** The statistical results of the user study for 10 different methods.

Metrics	Metric Values of Different Methods				
	DEM [11]	MEF-Net [14]	TransMEF [16]	DPE-MEF [19]	SwinFusion [17]
Total number	458	386	86	428	508
Preference rate	63.6%	53.6%	11.9%	59.4%	70.6%
Ranking	4	6	9	5	3
**Metrics**	**Metric Values of Different Methods**				
	**MEF-LUT [21]**	**UltraFusion [25]**	**GIFNet [27]**	**CDDFuse [18]**	**MEF-TBN**
Total number	78	526	194	316	**620**
Preference rate	10.8%	73.1%	26.9%	43.9%	**86.1%**
Ranking	10	2	8	7	**1**

In conclusion, the other methods have the disadvantage of inconsistent dynamic range, details lost or color distortion, but our method produces fusion images with the comparatively favorable visual quality.

### 4.2. Quantitative evaluation comparison

We conduct quantitative evaluation with quality assessment metrics presented by Zhang et al. [33] as shown in Table 2. We evaluated all the MEF methods with 10 representative metrics, including four different categories: information theory based (EN [35]), image feature based (AG [36], EI [37], SD [38], and SF [39]), image structural similarity based (Q^Y^ [40] and MEF-SSIM [13]), and human perception inspired (Q^CB^ [41], VIF [34] and NIQE [42]).

**Table 2:** Evaluation metrics used in this paper.

Metric Category	Metric Name	Meaning
Information theory based	EN [35]	Entropy
Image feature based	AG [36]	Average gradient
	EI [37]	Edge intensity
	SD [38]	Standard deviation
	SF [39]	Spatial frequency
Image structural similarity based	Q^Y^ [40]	Yang’s metric
	MEF-SSIM [13]	Multi-exposure fusion structural similarity index
Human perception inspired	Q^CB^ [41]	Chen-Blum metric
	VIF [34]	Visual information fidelity
	NIQE [42]	Natural Image Quality Evaluator

The clarity and detail performance of the fused image is measured by AG [36], which is higher for clearer edges and details and better quality. The amount of edge information that is preserved and enhanced in the fused image is measured by EI [37], which is higher for more edge information and better quality. The contrast and detail richness level of the fused image is measured by SD [38], which is higher for more details and contrast of the fused image. The detail clarity of the fused image is measured by SF [39] by calculating the gradient change in the horizontal and vertical directions. The higher values indicate the more details and edge information contained in the image. MEF-SSIM [13] is specially designed for the MEF task based on the structural similarity index (SSIM), which is higher for better fusion quality. Q^Y^ [40] is also based on the structural similarity (SSIM) combined with local contrast information of the image, which can effectively evaluate the performance of the fused image in detail preservation and contrast enhancement.

The above metrics focus on the image feature or structure similarity, but ignore the quality of human visual perception, such as color information. Q^CB^ [41] evaluates the image quality by simulating multiple human visual perception factors, including details, brightness, local contrast and colors, to generate an integrated quality score, which is higher for better quality. VIF [34] measures image quality by comparing the visual information fidelity between the fused image and the reference image based on the properties of the human visual system (HVS), which is higher for better quality of the fused image. NIQE [42] is a no-reference image quality assessment metric to evaluate the naturalness of the fused image based on human visual perception. The smaller values indicate better image quality.

Table 3–5 shows the average metric value ± standard deviation with 95% confidence intervals of different MEF methods for the testing dataset K=2, K=3 and K=5, respectively. The values highlighted in bold and underline in each table indicate them rank 1st and 2nd, respectively. The detailed metric values for all the sequences of all the comparison methods are presented in S2 Table of the Supporting Information.

**Table 3:** Average metric values ± standard deviation with 95% confidence intervals of different MEF methods for the testing dataset K=2.

Metrics	Metric Values of Different Methods				
	DEM [11]	MEF-Net [14]	TransMEF [16]	DPE-MEF [19]	SwinFusion [17]
EN [35]	7.4270 ± 0.0675	7.5232 ± 0.0880	5.7347 ± 0.1998	7.3812 ± 0.0583	7.2957 ± 0.0439
AG [36]	6.1549 ± 0.8244	6.7664 ± 0.8350	4.4518 ± 0.3688	6.9395 ± 0.9426	6.3757 ± 0.0856
EI [37]	60.1164 ± 7.2237	69.3748 ± 8.4422	34.8098 ± 4.8739	67.8272 ± 8.7238	64.0003 ± 5.0635
SD [38]	55.0990 ± 2.9048	62.2761 ± 3.3706	56.1788 ± 2.7048	60.8967 ± 2.2834	56.4825 ± 2.0475
SF [39]	20.2290 ± 2.0920	22.8203 ± 2.3584	13.1392 ± 1.0237	23.3471 ± 2.5320	20.4086 ± 1.0697
Q^Y^ [40]	**0.8622** ± 0.0216	0.8228 ± 0.0327	0.4854 ± 0.0178	0.7433 ± 0.0224	0.6544 ± 0.0126
MEF-SSIM [13]	0.9714 ± 0.0057	0.9471 ± 0.0147	0.8367 ± 0.0103	0.9332 ± 0.0331	0.9698 ± 0.0239
Q^CB^ [41]	0.5138 ± 0.0253	0.5036 ± 0.0280	0.4258 ± 0.0266	0.4055 ± 0.2111	0.4604 ± 0.0961
VIF [34]	0.8198 ± 0.0183	0.9209 ± 0.0252	0.4109 ± 0.0125	0.7125 ± 0.0213	0.7444 ± 0.0365
NIQE [42]	3.3477 ± 0.2789	3.3533 ± 0.2840	4.2553 ± 0.3026	3.5597 ± 0.2883	3.3279 ± 0.1437
**Metrics**	**Metric Values of Different Methods**				
	**MEF-LUT [21]**	**UltraFusion [25]**	**GIFNet [27]**	**CDDfuse [18]**	**MEF-TBN**
EN [35]	6.5257 ± 0.2099	**7.5806** ± 0.0810	6.1017 ± 0.1196	7.2001 ± 0.0535	7.5561 ± 0.0696
AG [36]	3.2789 ± 0.5161	6.9629 ± 0.6509	4.5681 ± 0.5986	6.9201 ± 0.3536	**7.1026** ± 0.8915
EI [37]	33.0644 ± 4.7359	67.0013 ± 8.0357	35.3319 ± 4.6645	68.5425 ± 5.0265	**72.1754** ± 8.6615
SD [38]	54.7640 ± 4.8641	62.3966 ± 2.5315	55.5315 ± 3.0142	60.4409 ± 2.0967	**63.8984** ± 3.7117
SF [39]	11.9486 ± 1.5977	23.1938 ± 1.9178	15.3236 ± 1.5133	23.1954 ± 1.4968	**23.6256** ± 2.2751
Q^Y^ [40]	0.5254 ± 0.0490	0.7384 ± 0.0143	0.6186 ± 0.0217	0.6556 ± 0.0273	0.8441 ± 0.0213
MEF-SSIM [13]	0.8242 ± 0.0271	**0.9743** ± 0.0030	0.8697 ± 0.0050	0.9381 ± 0.0037	0.9731 ± 0.0054
Q^CB^ [41]	0.4659 ± 0.0213	0.5131 ± 0.0337	0.4553 ± 0.2885	0.4238 ± 0.0637	**0.5286** ± 0.0191
VIF [34]	0.4188 ± 0.0516	0.9402 ± 0.0089	0.5418 ± 0.0528	0.8708 ± 0.0369	**0.9486** ± 0.0176
NIQE [42]	3.9021 ± 0.3394	3.5678 ± 0.3658	4.1476 ± 0.2331	3.3640 ± 0.1567	**3.3091** ± 0.2823

**Table 4:** Average metric values ± standard deviation with 95% confidence intervals of different MEF methods for the testing dataset K=3.

Metrics	Metric Values of Different Methods				
	DEM [11]	MEF-Net [14]	MEF-LUT [21]	SwinFusion [17]	MEF-TBN
EN [35]	7.3567 ± 0.1483	7.1673 ± 0.2241	5.9607 ± 0.6399	7.0268 ± 0.2687	**7.4280** ± 0.1381
AG [36]	6.9278 ± 1.3555	4.6604 ± 0.9290	3.2604 ± 0.3833	5.7982 ± 2.3476	**7.5372** ± 1.6173
EI [37]	68.6515 ± 13.0476	46.8231 ± 9.0084	33.2231 ± 3.7328	58.0304 ± 8.6352	**74.9991** ± 15.5045
SD [38]	59.1937 ± 4.5527	**60.7337** ± 4.5055	48.0671 ± 5.6800	60.2721 ± 4.6539	59.2452 ± 5.0013
SF [39]	22.0666 ± 3.4017	15.4117 ± 2.6128	11.6117 ± 1.1260	19.6385 ± 3.3857	**23.9144** ± 4.0999
Q^Y^ [40]	**0.8203** ± 0.0538	0.6505 ± 0.0444	0.4238 ± 0.0667	0.6926 ± 0.0369	0.7610 ± 0.0523
MEF-SSIM [13]	**0.9635** ± 0.0181	0.9176 ± 0.0257	0.8136 ± 0.0274	0.9105 ± 0.0216	0.9390 ± 0.0271
Q^CB^ [41]	0.4950 ± 0.0374	0.4586 ± 0.0371	0.4386 ± 0.0313	0.5106 ± 0.0229	**0.5146** ± 0.0338
VIF [34]	0.8099 ± 0.0569	0.6028 ± 0.0651	0.3895 ± 0.0615	0.7167 ± 0.0769	**0.8185** ± 0.0782
NIQE [42]	3.1410 ± 0.4317	3.3105 ± 0.4932	3.3088 ± 0.5294	3.3030 ± 0.5617	**3.0556** ± 0.4241

**Table 5:** Average metric values ± standard deviation with 95% confidence intervals of different MEF methods for the testing dataset K=5.

Metrics	Metric Values of Different Methods				
	DEM [11]	MEF-Net [14]	MEF-LUT [21]	SwinFusion [17]	MEF-TBN
EN [35]	7.4195 ± 0.0670	7.0110 ± 0.2459	5.5444 ± 0.2993	7.051 ± 0.0456	**7.4437** ± 0.0906
AG [36]	5.6480 ± 1.1486	3.2900 ± 0.5964	3.0900 ± 0.5242	5.322 ± 1.4281	**5.7774** ± 1.1844
EI [37]	57.7180 ± 11.2324	34.2704 ± 6.1348	30.6706 ± 3.9423	55.1937 ± 8.5647	**59.7489** ± 11.8851
SD [38]	57.6348 ± 4.6099	59.9653 ± 7.7321	47.2987 ± 2.9443	**64.3545** ± 5.3624	57.7007 ± 6.0710
SF [39]	18.4478 ± 3.0353	11.1589 ± 1.6046	10.2255 ± 1.4437	17.8541 ± 2.6581	**18.9940** ± 3.1066
Q^Y^ [40]	**0.7049** ± 0.0613	0.4487 ± 0.0536	0.3993 ± 0.0411	0.5101 ± 0.0628	0.6182 ± 0.0658
MEF-SSIM [13]	**0.9364** ± 0.0215	0.8591 ± 0.0350	0.7791 ± 0.0264	0.8921 ± 0.0357	0.9177 ± 0.0255
Q^CB^ [41]	0.4270 ± 0.0449	0.3568 ± 0.0322	0.3375 ± 0.0254	0.3847 ± 0.0694	**0.4381** ± 0.0379
VIF [34]	0.7738 ± 0.0586	0.5541 ± 0.0435	0.3394 ± 0.0362	0.7662 ± 0.0629	**0.7865** ± 0.0725
NIQE [42]	3.8238 ± 0.5574	4.3261 ± 0.7133	4.7510 ± 0.8873	3.7423 ± 0.4739	**3.6643** ± 0.5088

The information theory based metric EN [35] (K=3 and K=5), the image feature based metrics AG [36], EI [37], SF [39], SD [38] (K=2), and all the human perception inspired metrics Q^CB^ [41], VIF [34], NIQE [42] of our method are the best among all the comparison methods. Besides, EN [35] (K=2) and the image structural similarity based metrics Q^Y^ [40], MEF-SSIM [13] are the second best. 95% confidence intervals also show the dispersion of all the metrics to prove the stability and reliability of our results. Furthermore, Wilcoxon signed-rank tests are performed for K=3 and K=5 to determine whether the differences between our method and competing methods are statistically significant. Our method achieves significant improvement over the second best method DEM [11] with p-value < 0.05 for main metrics, including three human perception inspired metrics Q^CB^ (W=22), VIF (W=21) and NIQE (W=29) with the critical statistic value $W_{-}=\text{3}\text{5}$, confirming that the performance gains are meaningful rather than random fluctuations. This is mainly due to three advantages of our method. Firstly, the CAAN branch has strong local feature extraction ability but weak ability to capture long-distance dependency features, while the self-attention mechanism of transformer branch is specifically designed to capture global dependencies features, so our CAAN branch and transformer branch are able to extract both local and global features. Secondly, our merger module better integrates the local and global features extracted by the above two branches, and applies guided filtering for joint upsampling to upscale the low resolution weight maps to the original resolution for fusion. Thirdly, our color enhancement branch optimizes the appearance of the fused image by learning the correspondence between color and brightness, so as to significantly improve the quality of human visual perception with natural and vivid colors.

Compared with the state-of-the-art transformer-based method SwinFusion [17], our method achieves competitive performance on structural similarity metrics while maintaining better color consistency and visual naturalness, with about 11.5% increasement on Q^CB^ [41], 19.8% increasement on VIF [34], 2.2% improvement on NIQE [42] and 1.6% improvement on MEF-SSIM [13] for all the testing multi-exposure sequences.

For the image structural similarity based metric Q^Y^, our method is only lower than the structural block decomposition based method DEM [11], because DEM calculates the weight maps by decomposing each image block into mean intensity, signal strength and signal structure, which exactly improves the structural similarity and is highly consistent with the metric Q^Y^. However, DEM has the flaws of halo artifacts and color distortion. For the other image structural similarity based metric MEF-SSIM, our method is only lower than UltraFusion [25] because UltraFusion transforms the exposure fusion problem to a guided inpainting problem, and infers the fused image by the generative priors of the diffusion module. It decomposes the soft guidance underexposed image into the color and structure information, similar to SSIM as the structure component, which is further encoded using trained feature extractors. This strategy improves the structural features based on information theory, but weakens the constraint on color consistency. Though DEM [11] and UltraFusion [25] are better than our method for one image structural similarity based metric, our method is better than them for all the human perception inspired metrics Q^CB^ [41], VIF [34] and NIQE [42].

Above all, our method has high robustness for all the metrics, which is consistent with the subjective observation. The overview characteristics of the ten state-of-the-art MEF methods are also presented in Table 6, including the non-deep learning based method DEM [11] and nine other deep learning based methods. Compared with other methods, our method MEF-TBN is unsupervised with both CNN and Transformer based and color aware, and can support two or more input images, which also shows the advantages of our method.

**Table 6:** An overview characteristic of ten state-of-the-art MEF methods.

MEF Method	CNN based	Transformer based	Color aware	Supervised		Input Number Supported	
				Yes	No	K=2	K>2
DEM [11]	–	–	–	–	–		√
MEF-Net [14]	√				√		√
TransMEF [16]		√		√		√	
DPE-MEF [19]	√		√		√	√	
MEF-LUT [21]	√				√		√
UltraFusion [25]	√			√		√	
SwinFusion [17]		√		√			√
CDDFuse [18]	√	√		√		√	
GIFNet [27]	√				√	√	
MEF-TBN	√	√	√		√		√

### 4.3. Efficiency and computational complexity analysis

We also compare the efficiency and computational complexity of our method with other deep learning based methods, and compute the parameters, FLOPs, model size and average testing time for all compared methods. For fair comparison, we first record the average time on one NVIDIA GeForce RTX 2080 SUPER GPU and i7-12700 CPU for each sequence of the three testing datasets with K=2, K=3 and K=5, respectively, as shown in Table 7.

**Table 7:** The comparison of params, FLOPs, model size and average testing time for five methods.

MEF Method	Params (M)	FLOPs (G)	Model Size (MB)	Average Testing Time (s)		
				K=2	K=3	K=5
MEF-CAAN [15]	0.074	2.13	0.296	0.208	0.295	0.426
DPE-MEF [19]	13.6	16.5	54.4	0.492	0.624	1.382
SwinFusion [17]	34.2	41.5	136.8	1.852	2.786	3.214
UltraFusion [25]	217.0	126.3	868	105.9	185.5	298.3
MEF-TBN	23.5	26.7	94	0.644	0.754	1.667

The params of UltraFusion [25] are 217M, FLOPs are 126.3G, and model size is up to 868MB, which are much larger than other methods, so our MEF-TBN is significantly faster than UltraFusion [25]. The state-of-the-art transformer-based method SwinFusion [17] has 34.2M params, 41.5G FLOPs and 136.8MB model size, which also brings heavy computational overhead and low testing efficiency. The params of MEF-CAAN [15], DPE-MEF [19] and MEF-TBN are 0.074M, 13.6M and 23.5M, and their model sizes are 0.296MB, 54.4MB and 94MB, and their FLOPs are 2.13G, 16.5G and 26.7G, respectively. Although the efficiency of our three-branch network architecture is slightly lower than MEF-CAAN [15] and DPE-MEF [19], the quality of the fused images has significantly improved.

We also test the average time of each sequence on the testing dataset K=5 for each branch or module on GPU. The CAAN branch, the transformer branch, the color enhancement branch, the merger module and GFU take 0.128s, 0.641s, 0.255s, 0.187s and 0.394s, respectively. The results indicate that the CAAN branch, the color enhancement branch and the merger module are relatively efficient, taking up about 8%, 15% and 11% of the whole time, while the transformer branch and GFU are time-consuming, taking up about 38% and 24% of the whole time. The total average time is 1.667s and the remaining time is about 0.062s mainly used for initialization, color space conversion, and so on, taking up about 4% of the whole time.

### 4.4. Ablation studies

**Ablations on three branches.** Complete ablation experiments are conducted to demonstrate the effectiveness of each branch of MEF-TBN. We use the representative structural similarity based metric MEF-SSIM [13] and two human perception inspired metrics Q^CB^ [41] and VIF [34] as shown in Table 8. We also introduce Colorfulness Index [43] to evaluate the quality of our color enhancement branch. Colorfulness Index is an absolute metric that measures the overall vividness and saturation of colors based on the RGB color space of the fused image without relying on any reference, which is higher for better color quality with the value range [0, 100] under the sRGB color space. It can be observed that the average values of MEF-SSIM, Q^CB^ and VIF metrics are significantly decreased if any of the CAAN branch, transformer branch, color enhancement branch is removed. Besides, if the color enhancement branch is removed, the average value of Colorfulness Index is significantly decreased. Therefore, all the three branches are very important to improve the fusion quality.

**Table 8:** Average metric values of ablation experiments for three branches.

CAAN branch	Transformer branch	Color enhancement branch	MEF-SSIM	Q^CB^	VIF	Colorfulness Index
	√	√	0.9689	0.4997	0.9281	32.252
√		√	0.9678	0.5096	0.9378	41.681
√	√		0.9703	0.5152	0.9401	23.973
√	√	√	**0.9731**	**0.5286**	**0.9486**	**47.434**

**Fig 9:**
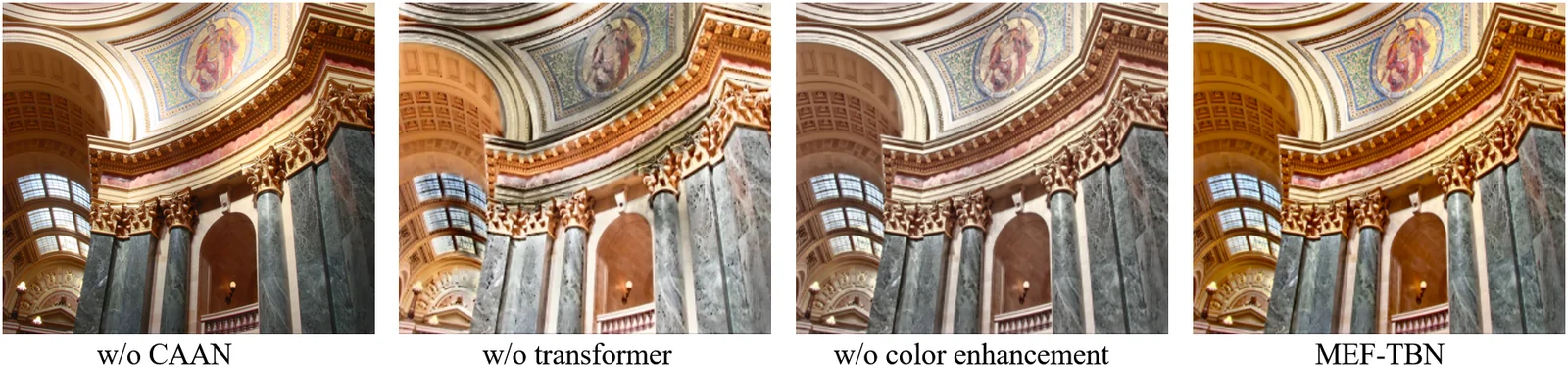
From left to right are the fusion results without CAAN branch, without transformer branch, without color enhancement branch and the full MEF-TBN, respectively.

The ablation experiments for three branches are also provided for subjective observation comparison in Fig 9. The two input images are $X_{\text{1}}$ and $X_{\text{2}}$ for the sequence ‘MadisonCapitol’ on the testing dataset K=2. Firstly, it can be observed that while the transformer branch deal with global exposure transition reasonably well, the fusion result without the CAAN branch has limitations in local underexposed or overexposed regions, where details and texture information are seriously lost. Secondly, the local details and textures are well preserved without the transformer branch because of the local feature extraction ability of CAAN, but the ability of global exposure transition is notably diminished. Thirdly, the results preserve vivid colors with the color enhancement branch, but the colors obtained by simple weighted summation strategy for each chrominance component appear significantly dull and distorted instead of our color enhancement branch, because the extreme exposed regions in the original images offer very limited color information. Finally, the fusion result generated by our complete three-branch network architecture successfully preserves rich detail information, vivid and natural colors, and achieves overall well-exposed visual perception quality.

**Fig 10:**
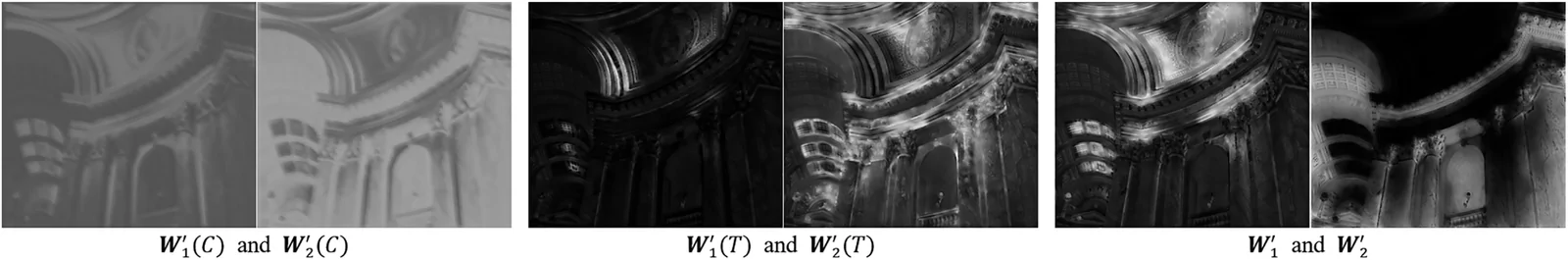
Visualizations of weight maps of CAAN branch, transformer branch and our full MEF-TBN.

We also visualize the learned weight maps on the sequence ‘MadisonCapitol’ in Fig 10, where a brighter pixel in $W_{k}^{\prime }$ indicates that the corresponding pixel in $X_{k}^{\prime }$ contributes more to the fused image. The weight maps of CAAN branch $\left\{W_{k}^{\prime }(C)\right\}$ focus on the local fine textures, such as outside the windows and the wall textures. The weight maps of transformer branch $\left\{W_{k}^{\prime }(T)\right\}$ focus on the long-distance features and global dynamic range, such as the whole exposure transition and large edges around the windows. The weight maps of our full MEF-TBN $\left\{W_{k}^{\prime }\right\}$ can fuse all the local details and global exposure features well.

**Ablations on merger module.** We further verify the effectiveness of our merger module. When $N_{b}=\text{0}$, the features extracted from the CAAN branch and the transformer branch are concatenated with simple element-wise addition to output the weight maps directly, without introducing the merger module. However, the metric values MEF-SSIM, Q^CB^ and VIF are only 0.9712, 0.5272 and 0.9454, respectively. In comparison, the full model with three merger module further improves these metrics to 0.9731, 0.5286 and 0.9486, respectively. The performance improvement demonstrates that the merger module with convolution-based refinement can effectively integrate and enhance fused features, which is indispensable and critical to the final fusion quality. When $N_{b}>\text{0}$, the merger module is introduced. As the number of merger blocks $N_{b}$ increases, the average values of all three metrics MEF-SSIM, Q^CB^ and VIF also increase accordingly as shown in Table 9. Therefore, the merger module is important to improve the fusion quality. However, when $N_{b}>\text{3}$, the values of all the three metrics increase slightly, such as MEF-SSIM value from 0.9731 to 0.9732, Q^CB^ value remains 0.5286, and VIF value from 0.9486 to 0.9487. Therefore, the block number of our merger module is set to 3 for balancing quality and efficiency.

**Table 9:** Average metric values for different numbers of merger blocks.

The number of merger blocks	MEF-SSIM [13]	Q^CB^ [41]	VIF [34]
0	0.9712	0.5272	0.9454
1	0.9721	0.5283	0.9481
2	0.9727	0.5285	0.9484
3	0.9731	0.5286	0.9486
4	0.9732	0.5286	0.9487
3 (with cross-attention based fusion)	0.9711	0.5138	0.9026

**Fig 11:**
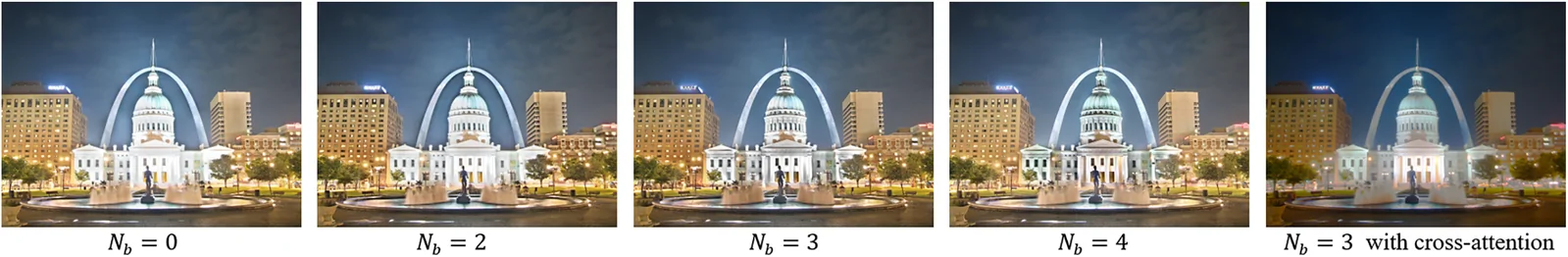
The fusion results with different numbers of merger blocks.

The ablation experiments for different numbers of merger blocks are provided for subjective observation comparison in Fig 11 on the sequence ‘Night’. It can be observed that as the number of merger blocks increases, the details and texture information in the underexposed and overexposed regions are preserved better, especially on the overexposed building surface. However, when the block number is larger than 3, there is very little improvement in visual perception, which is highly consistent with the objective measurement.

We also introduce the cross-attention based fusion strategy like SwinFusion [17] for the $N_{b}=\text{3}$ model instead of our convolution-based refinement, but the metric values MEF-SSIM, Q^CB^ and VIF are down to 0.9711, 0.5138 and 0.9026, respectively. The cross-attention based fusion strategy also lead to insufficient local detail preservation and over-smoothing artifacts, as shown in Fig 11. Although the attention mechanism is proved to be effective for feature extraction stage that has been applied in our CAAN branch, it has some disadvantages for the fusion stage. The attention mechanism may allocate inappropriate weights to salient target regions and the background regions, causing information loss of effective target features. Besides, the attention mechanism overemphasizes on long-range dependencies for smoothing small-scale structural details in the final fused image. Our convolution-based merger module is more efficient, and effectively avoids over-smoothing artifacts to ensure the feature fusion ability.

The ablation experiments demonstrate that our merger module with convolution-based refinement plays an important role for features integration from the CAAN branch and transformer branch, and better than the other attention based fusion methods.

**Fig 12:**
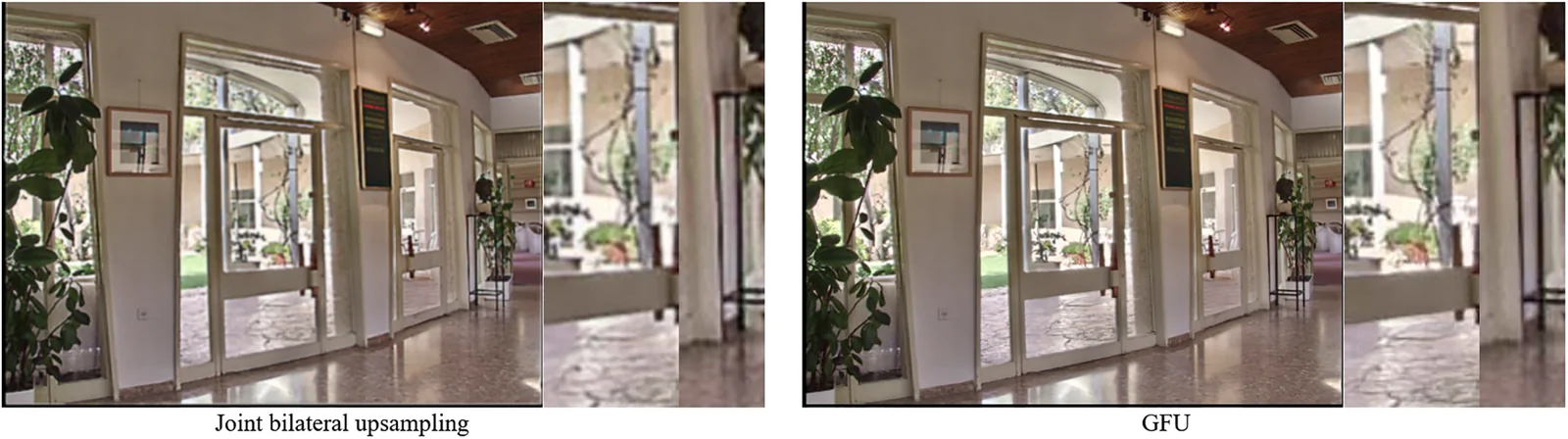
The fusion results and the close-up by joint bilateral upsampling and by our GFU.

**Ablations on different upsampling modules.** We also conduct ablation study on the upsampling module by comparing our guided filtering for upsampling (GFU) with joint bilateral upsampling [44]. The average MEF-SSIM [13] value with joint bilateral upsampling is 0.9682, about 0.005 lower than that with GFU (0.9731). Besides, the efficiency of GFU is much faster than joint bilateral upsampling because GFU adopts several linear operations instead of complex exponential operations. The results of two different upsampling ways are shown in Fig 12 for the testing sequence ‘BelgiumHouse’. Significant artifacts on the edge or distortions are introduced by joint bilateral upsampling while our GFU achieves comparable visual quality with lower computational cost. Therefore, this demonstrates the effectiveness of GFU module.

We have conducted additional ablations on the guided filter parameters radius r and regularization parameter ε as shown in Table 10. The larger parameters values can generate smooth weight maps but lose fine details, leading to decrease of MEF-SSIM values, but the smaller values may introduce noise or artifacts. The ablation results show that the optimal performance is obtained for balancing detail preservation and noise suppression when the radius r and regularization parameter ε are set to 1 and ${\text{10}}^{-\text{4}}$, respectively.

**Table 10:** Average MEF-SSIM values for different guided filter parameters.

radius r	1	2	4	8	regularization parameter ε	$10^{-1}$	$10^{-2}$	$10^{-4}$	$10^{-8}$
**MEF-SSIM**	**0.9731**	0.9720	0.9686	0.9634	**MEF-SSIM**	0.9711	0.9719	**0.9731**	0.9698

**Ablations on resolution, depth and width of CAAN branch.** Our default input low and high resolution of our training network are 256s and 512s, respectively, with the resolution conversion factor 2. We maintain the high resolution unchanged, and conduct ablations for training different low resolution from 32s to 256s with the conversion factor from 16 to 2, as shown in Table 11. We observe that the average MEF-SSIM value has improved from 32s to 256s, and the most satisfactory performance is obtained for training the low resolution 256s.

**Table 11:** Average MEF-SSIM values for training different low resolution.

Training low resolution	32	64	128	256
**MEF-SSIM**	0.9576	0.9661	0.9703	**0.9731**

Although our network is trained on the high resolution of 512s, it also achieves satisfactory results for input testing images with much higher resolution. We downsample 15 testing sequences to four kinds of resolutions from 512s to 2048s, with the average MEF-SSIM values shown in Table 12. We can observe that all the results are remarkably well, and from 512s to 2048s the MEF-SSIM value decreased only about 0.001, which proves that our method has high adaptability to testing images with higher resolution.

**Table 12:** Average MEF-SSIM values for different resolution input images.

Input resolution	512	1024	1536	2048
**MEF-SSIM**	0.9733	0.9730	0.9726	0.9722

We also perform ablation study on the dilation pattern of the CAAN branch. The average MEF-SSIM values are listed in Table 13, with increase as the depth and width of the CAAN branch increase. It can also be observed that the MEF-SSIM values reach more than 0.973 with the depth 6 or the width 24, but do not improve significantly with more depth or width, so the depth and width of the network of CAAN branch are set to 6 and 24 for higher efficiency performance.

**Table 13:** Average MEF-SSIM values for different network depth and width.

Depth	4	5	6	7	8	Width	8	16	24	32	48
**MEF-SSIM**	0.9684	0.9714	0.9731	0.9738	0.9742	**MEF-SSIM**	0.9588	0.9697	0.9731	0.9736	0.9740

### 4.5. Limitation in dynamic scenes

Our method has achieved very high quality for the static scenes, but will cause ghost artifacts when dealing with all the dynamic scenes with misaligned objects or moving objects. It is challenging for the current framework to deal with dynamic scenes because it lacks an effective motion detection mechanism or image alignment mechanism to automatically correct pixel displacements across multi-exposure images. For example, Demirbilek et al. [45] proposed the two-stage dense alignment method based on RANSAC-flow with a CNN merging network for ghost-free HDR imaging of dynamic scenes. We will also consider integrating such dedicated alignment or deghosting module into our MEF-TBN method in the future.

## 5. Conclusions

In this paper, we propose MEF-TBN with a three-branch network for multi-exposure image fusion task of static scenes, including the CAAN branch, the transformer branch and the color enhancement branch. The CAAN branch and the transformer branch fully utilize the local feature extraction ability of the convolutional neural network and the global long-distance dependency modeling ability of transformer, combining the advantages of both. The proposed merger module can adaptively integrate features extracted from both the CAAN branch and transformer branch, and apply the guided filtering for joint upsampling to upscale the low-resolution weight maps to the original resolution. Our color enhancement branch can optimize the appearance of the fused image to achieve more natural colors by learning the relationship between color and luminance in the same input image. In summary, our method has obvious advantages on details and colors preserving and visual perceptual quality improvement, and has acceptable time efficiency due to our feature extraction network in low-resolution space. Besides, our method has high robustness for sequences of static scenes with arbitrary spatial resolution and arbitrary number of exposures.

In the future, we would like to explore more effective feature extraction strategies and more visual perceptual consistency loss functions that are more consistent with human visual perception. We also consider more lightweight hierarchical transformers as our transformer branch network to further enhance the time efficiency of our method. Since our current method is only applicable to static scenes, we also consider introducing more ghost removal mechanisms to our network to adapt to dynamic scenes, such as motion masks, optical flow, diffusion-based alignment, and so on.

## Supporting information

S1 Table Sequences of training and testing dataset. (XLSX)

S2 Table Metrics values of all the sequences. (XLSX)
